# Retraction: Overexpression of Rad51 Predicts Poor Prognosis in Colorectal Cancer: Our Experience with 54 Patients

**DOI:** 10.1371/journal.pone.0206398

**Published:** 2018-11-01

**Authors:** The PLOS ONE Editors

After publication of this article [1], it came to light that the authors did not have prospective ethical approval for the reported study which involved patient data and clinical samples. Hence, this work did not comply with *PLOS ONE*’s requirements for human subjects research.

In light of the lack of ethical approval, the *PLOS ONE* Editors retract this article.

In addition, concerns were raised about overlap between this article [1] and an earlier publication by another group in the *International Journal of Clinical and Experimental Pathology* (hereafter, *IJCEP*; [2]). Tables 1, 2, and Figs 2, 3, and 4 in the *PLOS ONE* article present the same results as Tables 1, 2, and Figs 1, 2, and 3 of the *IJCEP* article. These articles address the same research question and report the same data, results, and conclusions, although the research in each case is reported as done by different research groups using samples collected in different periods and at different institutions.

We discussed the overlap concerns with the authors of both articles, and this was also investigated by Sichuan University. The *PLOS ONE* authors provided primary data files and noted that after completion of the study they obtained assistance with their manuscript from an external consulting company. An institutional representative affirmed these statements. The *IJCEP* authors commented that a third-party company had done some experiments for their article and had not provided the raw data. The authors of the *IJCEP* article retracted that publication [3] in 2018 due to errors in survival data in a figure that overlaps with the *PLOS ONE* article, and concerns about the validity of the conclusions.

YL, WyW, JhX, FX, DyL, LX, JW, and FL did not agree with retraction.
